# Chromosome-level genomes of hard clams *Meretrix lamarckii* (Deshayes, 1853) and *Meretrix meretrix* (Linnaeus, 1758)

**DOI:** 10.1038/s41597-026-07119-0

**Published:** 2026-03-27

**Authors:** Sean Tsz Sum Law, Wenyan Nong, Ming Fung Franco Au, Leni Hiu Tung Cheung, Cheryl Wood Yee Shum, Shing Yip Lee, Siu Gin Cheung, Jerome Ho Lam Hui

**Affiliations:** 1 School of Life Sciences, Simon F.S. Li Marine Science Laboratory, Institute of Environment, Energy and Sustainability, State Key Laboratory of Agrobiotechnology, The Chinese University of Hong Kong, Hong Kong SAR, China; 2 Simon F.S. Li Marine Science Laboratory, School of Life Sciences, The Chinese University of Hong Kong, Hong Kong SAR, China; Australian Rivers Institute, Griffith University Gold Coast campus, Southport, Qld 4222 Australia; 3 Department of Chemistry, State Key Laboratory of Marine Pollution, City University of Hong Kong, Hong Kong SAR, China

**Keywords:** Zoology, Evolution

## Abstract

The genus *Meretrix* contains marine bivalve molluscs commonly known as the Venus clams or hard clams, which can be found in the estuarine and marine habitats in Asia. Given their edibility, they have been exploited in clam digging activities and also been farmed in some places. Here, we provide two new high-quality genomes of species *M. meretrix* and *M. lamarckii*. Utilising a combination of PacBio HiFi and Omni-C sequencing technologies, genome assemblies of *M. meretrix* and *M. lamarcki* are obtained with sizes 835.1 Mb (scaffold N50 = 46 Mb) and 890.5 Mb (scaffold N50 = 46 Mb), respectively. More than 99% of sequences were anchored to 19 pseudochromosomes, and high completeness was also obtained estimated by BUSCO scores (~99.5%, mollusca_odb12). The two new genomic resources provided in this study will be useful for further understanding biology, ecology, and evolution of edible clams.

## Background & Summary

The family Veneridae contains more than 700 species of estuarine and marine bivalves worldwide. Species in the genus *Meretrix* are widely distributed in the coastal waters of the Indo-Pacific region^1^, and some of them are economically significant in aquaculture industry in the East and Southeast Asia^1–3^. Natural populations of *Meretrix* spp. are also susceptible to anthropogenic activities such as over-harvesting at industrial scale, recreational clam-digging and habitat degradation^2,4,5^.

Among the 17 described *Meretrix* species^6^, some of them have high morphological similarities, and molecular analyses are required for identification of distinct populations in different geographical locations^3,7,8^. For instance, a distinct clade (and later become a new species) of *M. taiwanica* has been detected from COI barcoding sequences^7,9^, and utilization of microsatellite DNA markers have revealed subpopulations of *M. meretrix*^3^. Genomic resources of *Meretrix* are thus important to understand and reveal the diversity between species and populations. As of to date, a total of four *Meretrix* genomes are available including *M. petechialis*^10^, *M. lamarckii*^7^, and two distinct *Meretrix* sp. MF1 and *Meretrix* sp. MT1^7^. Here, we present two new chromosomal-level genomes of *M. meretrix* and *M. lamarckii* for the further investigation of genetic diversity and of evolutionary of *Meretrix* clams.

## Methods

### Sample collection, DNA extraction and Species identification

Clams were collected in Tung Wan of Lantau Island in Hong Kong on 13 August 2022. Genomic DNA was extracted from the adductor muscle using Purelink Genomic Mini Kit (Invitrogen) following the manufacturer’s instructions. The quality and quantity of the isolated DNA was assessed by gel electrophoresis and NanoDrop spectrophotometer (Thermo Scientific). The species identity of the specimen was determined by the amplification of mitochondrial DNA cytochrome oxidase subunit I (COI) gene in polymerase chain reaction (PCR) using the universal COI primers LCO1490 and HCO2198^11^. The PCR was performed on a T100^TM^ thermocycler (Bio-Rad) with the following parameters: 95 °C for 3 minutes, followed by 39 cycles of 30 seconds at 95 °C, 30 seconds at 55 °C, and 45 seconds at 72 °C, and a final extension step at 72 °C for 5 minutes. The fragment size of amplified product was inspected on gel electrophoresis and was sent to BGI Hong Kong for Sanger sequencing. The chromatograms of the sequences were checked on Chromas v2.6.6 (https://technelysium.com.au/wp/chromas/) for quality control. Sequences were searched for homology in NCBI nr database using BLASTn. Subsequently, the sequences were aligned with reference COI sequences from previous studies^12–14^ using MAFFT^15^. A maximum likelihood phylogenetic tree was constructed using FastTree^16^.

### High molecular weight DNA extraction and long read sequencing

~300 mg of gill and adductor muscle tissues from *M. meretrix* and *M. lamarckii* respectively were ground into powder under liquid nitrogen. High molecular weight DNA was then isolated from the powder using the NucleoBond HMW DNA kit (Macherey-Nagel) following the manufacturer’s instructions. The resulting DNA was subjected to quality and quantity measurement using NanoDrop spectrophotometer (Thermo Scientific), Qubit® Fluorometer and pulse-field gel electrophoresis. The qualified DNA samples were sent to Macrogen for PacBio high-fidelity (HiFi) sequencing on the PacBio Revio system. 62.87 Gb and 57.89 Gb of HiFi reads were generated for *M. meretrix* and *M. lamarckii*, respectively (Table 1).

**Table 1:** Genome sequencing data of *M. meretrix* and *M. lamarckii*.

Species	Samples	Reads	Bases	Hifi average length (bp)	NCBI/ENA SRA Accesion	DDBJ DRA Accession	NGDC GSA Accession	CUHK Research Data Repository
*Meretrix meretrix*	Mmer_PacBio	4,376,435	62,875,654,323	14,367	SRR35263659	DRR911404	CRR2744619	10.48668/DOVLYS
*Meretrix meretrix*	Mmer_OmniC	23,697,482	3,554,622,300	—	SRR35263658	DRR911405	CRR2744620	
*Meretrix lamarckii*	Mlam_PacBio	4,402,942	57,891,566,865	13,148	SRR35291242	DRR911157	CRR2744623	
*Meretrix lamarckii*	Mlam_OmniC	22,436,556	3,365,483,400	—	SRR35291243	DRR911158	CRR2744624	

### Omni-C library preparation and sequencing

Omni-C libraries of *M. meretrix* and *M. lamarckii* were constructed from ~100 mg of gill tissues using the Dovetail® Omni-C® Library Preparation Kit (Cantata Bio) following the manufacturer’s instructions. The fragment size distribution and concentration of the resulting libraries were assessed with Agilent D5000 ScreenTape Assay and Qubit® Fluorometer. The qualified libraries were sent to Novogene for sequencing on an Illumina PE150 platform to obtain 3.55 Gb and 3.36 Gb Omni-C data for *M. meretrix* and *M. lamarckii*, respectively (Table 1).

### Genome assemblies

**Fig. 1:**
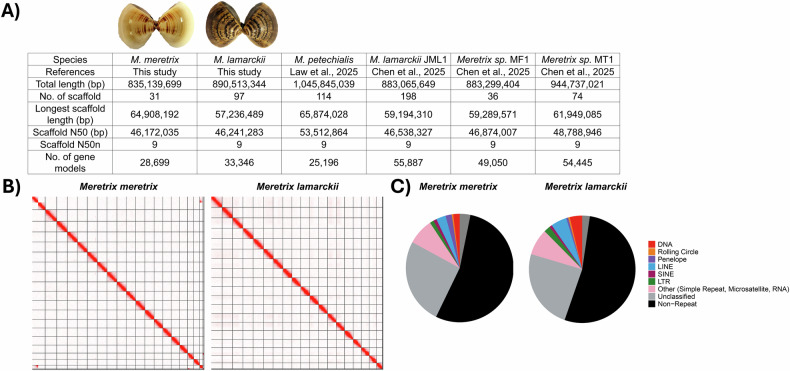
(**A**) Summary of genome assemblies of *M. meretrix* and *M. lamarckii* and other published *Meretrix* genomes. Scaffold N50n refers to the number of scaffolds that contribute to the N50 length; (**B**) Omni-C contact maps of the genome assemblies of *M. meretrix* (left) and *M. lamarckii* (right); **C)** Pie charts of annotated transposable elements of *M. meretrix* (left) and *M. lamarckii* (right).

*De novo* genome assemblies of *M. meretrix* and *M. lamarckii* were performed with Hifiasm^17^. Haplotypic duplications were further removed using *purge_dups*^18^. The contigs of the draft assemblies were searched against the NT database using BLASTn to filter out potential contaminations with BlobTools (v1.1.1)^19^. Subsequently, the assemblies were scaffolded with the Omni-C data using YaHS^20^ and manually checked using Juicebox (v1.11.08)^21^. Finally, the assembled genome sequences were assessed with NCBI Foreign Contamination Screen (FCS)^22^ to remove contaminant sequences. The final assemblies of *M. meretrix* and *M. lamarckii* were 853.1 Mb and 890.5 Mb in size, and 99.04% and 99.43% of the sequences were anchored to 19 chromosomal scaffolds, respectively (Fig. 1A,B; Tables 2, 3). Both *Meretrix* genomes were of high sequence continuity with scaffold N50 of ~46.2 Mb (Table 2).

**Table 2:** Genome statistics of *M. meretrix* and *M. lamarckii* and other published *Meretrix* genomes.

Species	Meretrix meretrix	Meretrix lamarckii	Meretrix petechialis	M. lamarckii JML1	Meretrix sp. MF1	Meretrix sp. MT1
References	This study	This study	Law *et al*., 2025	Chen *et al*., 20205	Chen *et al*., 20205	Chen *et al*., 20205
Total length	835,139,699	890,513,344	1,045,845,039	883,065,649	883,299,404	944,737,021
No. of scaffold	31	97	114	198	36	74
Mean scaffold length	26,939,990	9,180,550	9,174,079	4,459,928	24,536,095	12,766,717
Longest scaffold length	64,908,192	57,236,489	65,874,028	59,194,310	59,289,571	61,949,085
Gaps	14	33	198	0	0	0
Scaffold N50	46,172,035	46,241,283	53,512,864	46,538,327	46,874,007	48,788,946
Scaffold N50n	9	9	9	9	9	9
No. of gene models	28,699	33,346	25,196	55,887	49,050	54,445
No. of longest-transcripts	26,759	30,829	20,084	51,779	45,263	50,420

**Table 3:** Chromosome information of *M. meretrix* and *M. lamarckii*.

Chr number	Chr ID	Meretrix meretrix		Meretrix lamarckii	
		Chr length (bp)	% of the whole genome	Chr length (bp)	% of the whole genome
1	scaffold_1	64,908,192	7.77	57,236,489	6.43
2	scaffold_2	54,483,009	6.52	54,346,355	6.10
3	scaffold_3	51,357,909	6.15	53,604,160	6.02
4	scaffold_4	49,509,254	5.93	53,479,476	6.01
5	scaffold_5	48,075,356	5.76	52,294,165	5.87
6	scaffold_6	47,988,444	5.75	51,023,760	5.73
7	scaffold_7	47,650,749	5.71	49,992,336	5.61
8	scaffold_8	47,076,688	5.64	46,475,248	5.22
9	scaffold_9	46,172,035	5.53	46,241,283	5.19
10	scaffold_10	45,271,054	5.42	46,212,281	5.19
11	scaffold_11	45,158,256	5.41	46,002,274	5.17
12	scaffold_12	43,632,693	5.22	45,873,465	5.15
13	scaffold_13	42,715,300	5.11	44,241,166	4.97
14	scaffold_14	42,254,144	5.06	43,993,078	4.94
15	scaffold_15	39,053,538	4.68	43,461,710	4.88
16	scaffold_16	38,933,291	4.66	42,595,028	4.78
17	scaffold_17	29,759,200	3.56	41,225,765	4.63
18	scaffold_18	27,822,048	3.33	37,866,541	4.25
19	scaffold_19	15,296,840	1.83	29,279,834	3.29
	**sum=**	827,118,000	99.04	885,444,414	99.43

### Genome annotation

Gene model prediction was performed using Braker (v3.0.8)^23^ with default parameters. Protein hints with 389,399 Mollusca reference protein sequences downloaded from NCBI were used to train GeneMark-EP + without RNA-Seq data, whereas genes with high extrinsic evidence support were subsequently used to train AUGUSTUS^24^. A total of 28,699 and 33,346 gene models were predicted from the *M. meretrix* and *M. lamarckii* genomes, respectively (Table 2).

### Repeat annotation

Transposable elements (TEs) of the two genomes were annotated using Earl Grey (v1.2)^25^ with the option “-r eukarya” specified for the initial mask of known TEs within the Earl Grey automated pipeline. In total, 357.2 Mb and 397.5 Mb of repetitive elements were identified in the genomes of *M. meretrix* and *M. lamarckii*, which account for 42.77% and 44.64% of respective genome (Fig. 1C; Table 4).

**Table 4:** Repeat annotations of *M. meretrix* and *M. lamarckii*.

Classification	Coverage (bp)	Count	Proportion (%)	No. of distinct classifications	Coverage (bp)	Count	Proportion (%)	No. of distinct classifications
	*Meretrix meretrix*				*Meretrix lamarckii*			
DNA	16,308,484	57,727	1.95	5,814	33,779,114	120,855	3.79	14,768
LINE	24,165,708	78,322	2.89	8,606	43,085,090	138,412	4.84	16,107
LTR	9,914,756	28,900	1.19	5,758	17,627,897	43,593	1.98	9,100
Other (Simple Repeat, Microsatellite, RNA)	65,235,986	301,859	7.81	4,627	70,941,785	331,613	7.97	5,672
Penelope	15,486,324	33,740	1.85	5,542	5,276,681	9,932	0.59	3,996
Rolling Circle	3,717,496	12,133	0.45	3,697	4,236,078	9,895	0.48	3,343
SINE	8,010,153	41,567	0.96	9,559	7,064,880	42,137	0.79	5,763
Unclassified	214,347,055	884,731	25.67	10,742	215,536,269	829,761	24.20	14,463
Sum	357,185,962	1,438,979	42.77	54,345	397,547,794	1,526,198	44.64	73,212

### Phylogenomic tree analysis

**Fig. 2:**
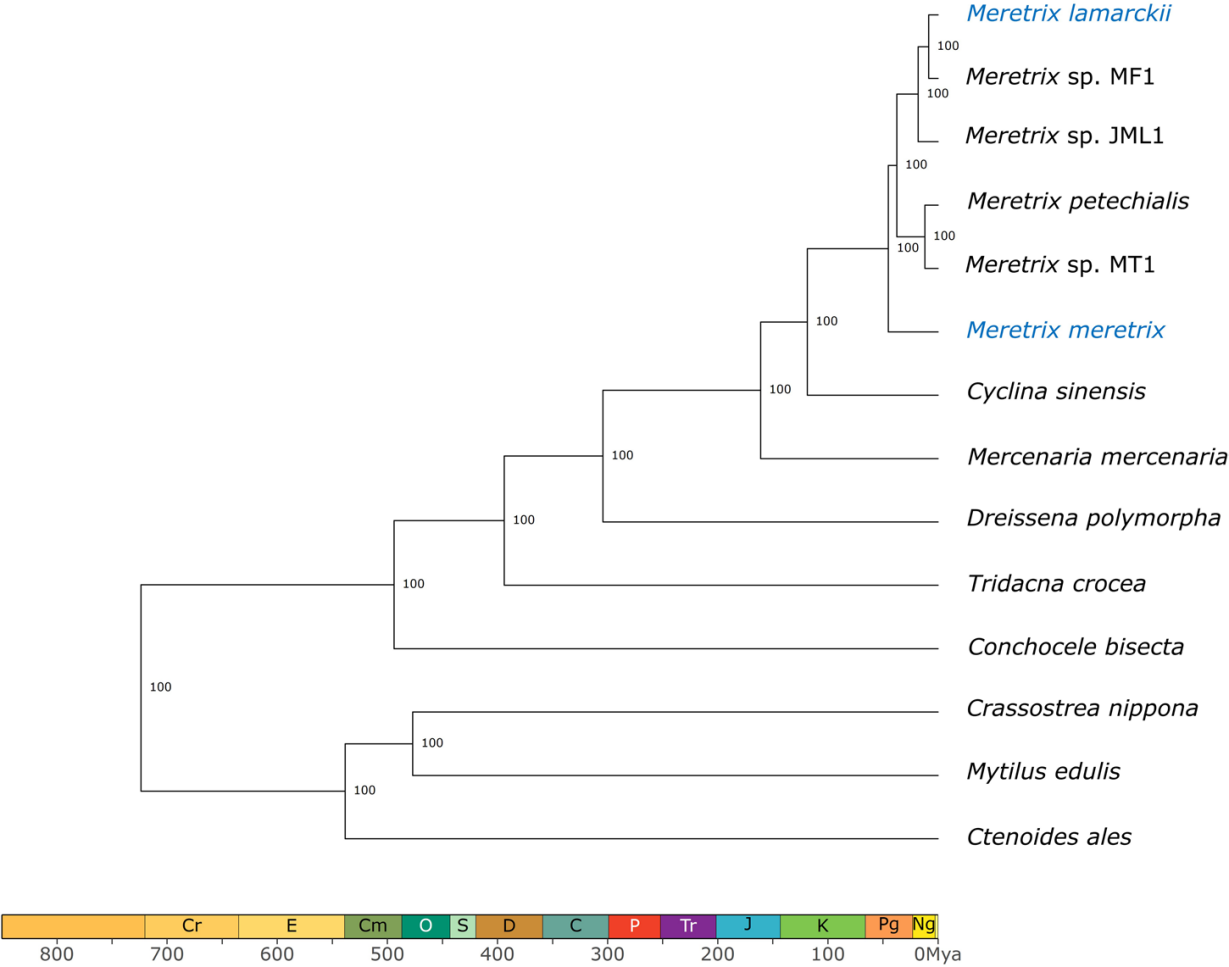
Phylogenomic tree of *Meretrix* species and 8 other bivalve species. Mya: million years ago.

To elucidate the phylogenetic relationships between the two genomes generated in this study and other *Meretrix* genomes, gene orthology analysis was carried out from the longest transcript of proteome together with 8 other bivalve genomes using OrthoFinder v. 2.5.2^26^. These genomes include *M. petechialis* (GCA_046203225.1)^10^, *Meretrix* sp. JML1 (GCA_049244375.1), *Meretrix* sp. MF1 (GCA_049244355.1) and *Meretrix* sp. MT1 (GCA_049244365.1)^7^, *Cyclina sinensis* (GCA_012932295.1)^27^, *Mercenaria mercenaria* (GCA_014805675.2)^28^, *Dreissena polymorpha* (GCF_020536995.1)^29^, *Tridacna crocea* (GCA_032873355.1)^30^, *Conchocele bisecta* (GCA_029237695.1)^31^, *Crassostrea nippona* (GCA_033439105.1)^32^, *Mytilus edulis* (GCF_036588685.1)^33^ and *Ctenoides ales* (GCA_042257175.1)^34^. 1,210 single-copy orthologs were further used for the construction of a phylogenomic tree in the automated OrthoFinder pipeline, which employs the STAG and STRIDE algorithms for species tree inference and tree rooting, respectively^26^. The resulting tree was used as input to estimate the divergence time using r8s^35^, with a prior divergence time estimate of 494 million years ago between *Conchocele bisecta* and *Tridacna crocea* adapted from the TimeTree database^36^. The resulting phylogenomic tree revealed that *M. meretrix* is sister to other sequenced *Meretrix* genomes (Fig. 2).

## Technical Validation

### Sequence quality

**Fig. 3:**
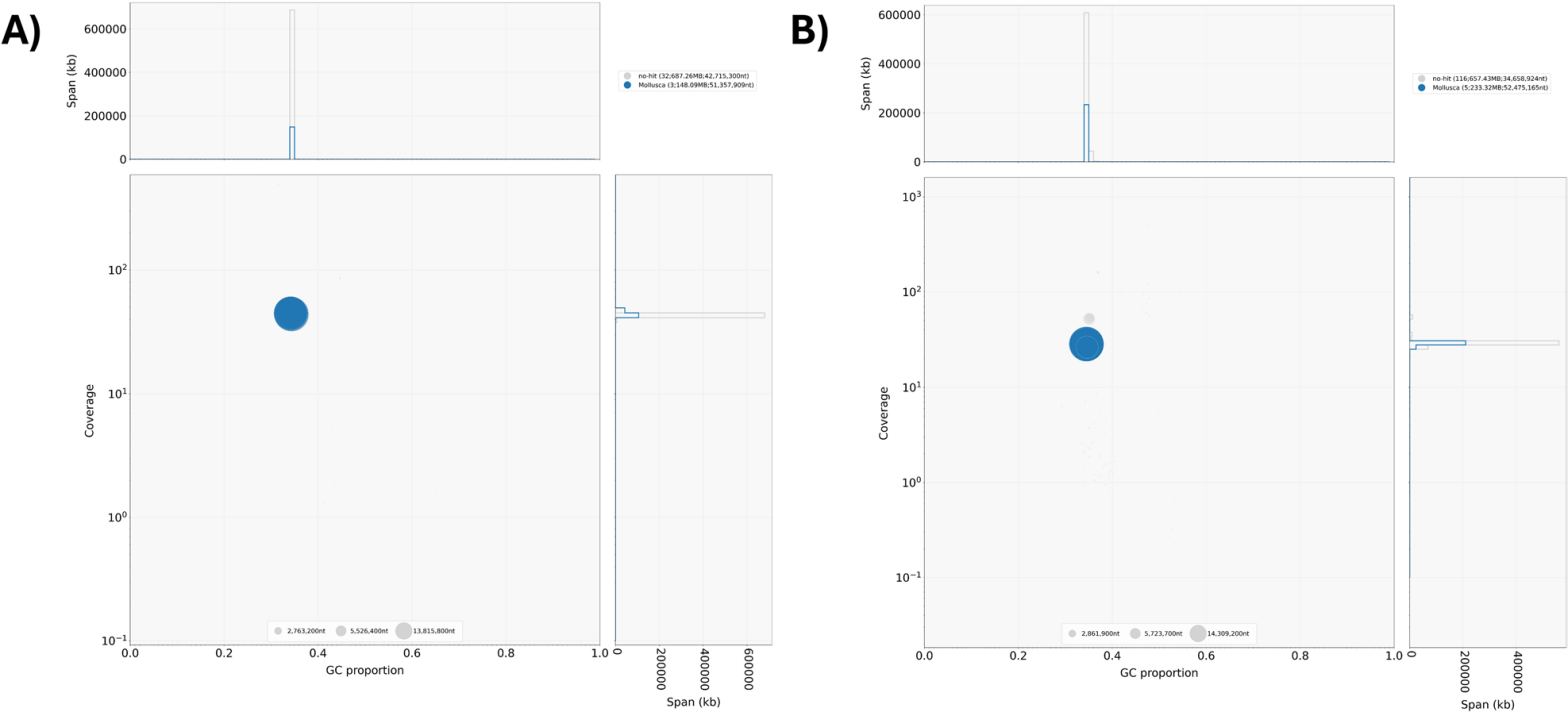
Blobtools GC-coverage plots for the draft genome assembly control prior to scaffolding for *M. meretrix* (**A**) and *M. lamarckii* (**B**). The size of the circles are proportional to the contig length.

Prior to genome assembly scaffolding, the contigs in the draft assemblies were screened for potential contaminations with BlobTools^19^. All 35 and 121 contigs in the draft assemblies of *M. meretrix* and *M. lamarckii* were affiliated with taxonomic class “Mollusca” or unknown taxonomic class (“no-hit”) and were retained for scaffolding, respectively (Fig. 3). After scaffolding with Omni-C data, NCBI Foreign Contamination Screen (FCS)^22^ was used for detecting potential contaminated scaffolds in the genome assemblies (Supplementary Table S1). Out of 37 scaffolds in the *M. meretrix* genome assembly, 6 scaffolds were affiliated as prokaryotes, accounting for 214,586 bp in total. On the other hand, of the 102 scaffolds in the *M. lamarckii* genome assembly, 5 scaffolds and 1 scaffold were identified as prokaryotic and mitochondrial sequences, accounting for 239,857 bp and 23,565 bp, respectively. Subsequently, the identified contaminated scaffolds were searched against the respective genome assemblies using BLASTn. While no blast hits of contaminated scaffolds were found in the remaining scaffolds of the *M. meretrix* assembly, 8 scaffolds and 1 scaffold in the *M. lamarckii* assembly were detected for blast hits against two of the prokaryotic contaminated scaffolds and the mitochondrial scaffold, respectively (Supplementary Table S1). Manual BLASTn searches on NCBI core_nt database were performed for the potential contaminated scaffolds, from which 0%–2% query coverages resulted for prokaryotic contaminated scaffolds and a 100% query coverage was matched with the *M. lamarckii* mitochondrial genome (accession NC_016174.1) for the mitochondrial scaffold. Therefore, the prokaryotic contaminated scaffolds identified by FCS were removed from the final assemblies and the remaining potential contaminated scaffolds were documented in Supplementary Table S1.

### Evaluation of genome assembly and annotation completeness

**Fig. 4:**
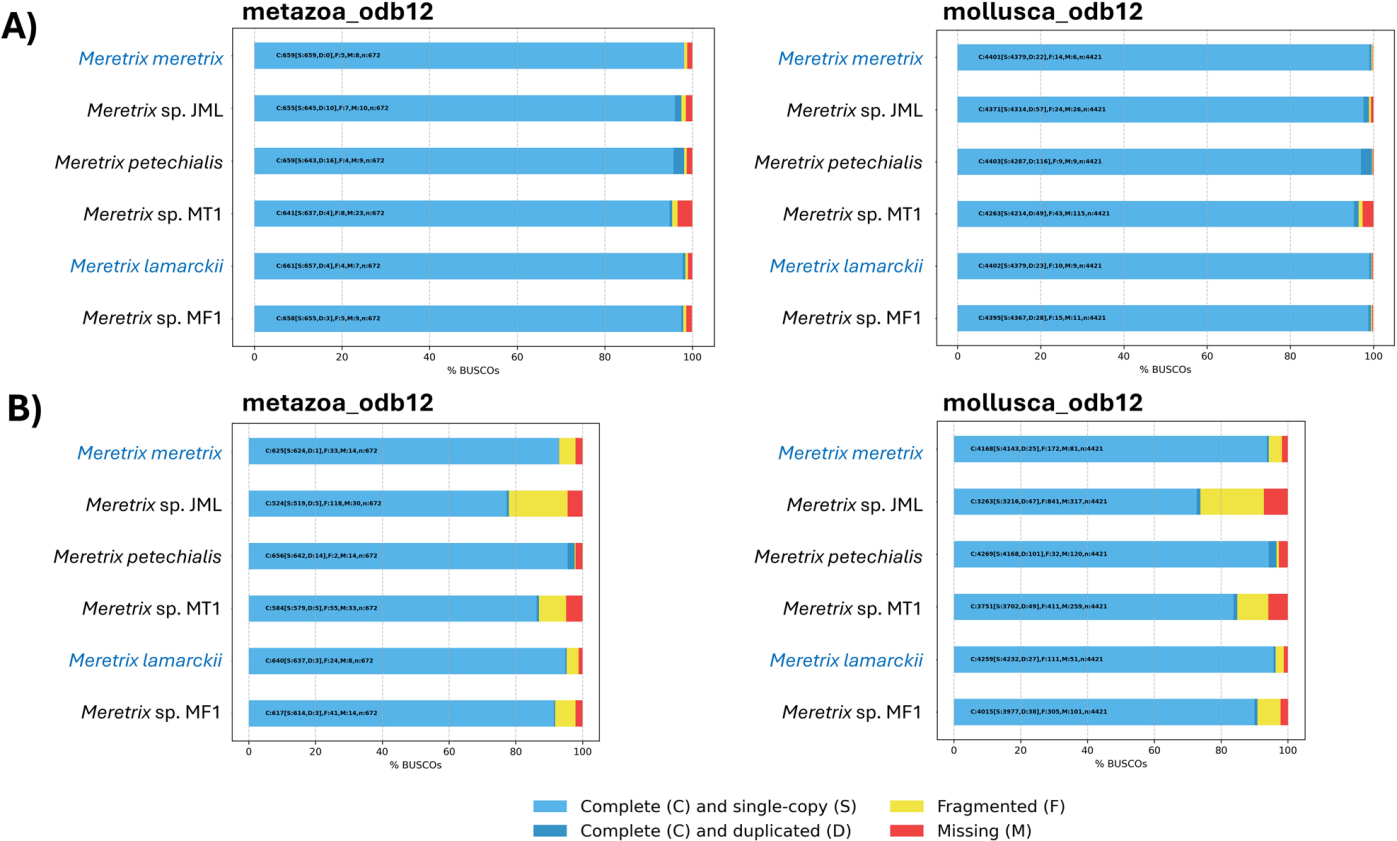
BUSCO completeness assessment of the (**A**) genome assemblies and (**B**) predicted gene models of *M. meretrix* and *M. lamarckii* and other published *Meretrix* genomes using the metazoa_odb12 database (left) and mollusca_odb12 database (right).

The completeness of the genome assemblies and annotations of *M. meretrix* and *M. lamarckii* were assessed with BUSCO (v6.0.0)^37^ using databases metazoa_odb12 and mollsuca_odb12, respectively, and were compared to other available *Meretrix* genomes (Fig. 4). Out of 672 single-copy orthologs in the metazoa_odb12 database, 659 (98.1%) and 661 (98.4%) single-copy orthologs were identified in the genome assemblies of *M. meretrix* and *M. lamarckii*, respectively (Fig. 4A). In addition, almost complete sets of single-copy orthologues from the mollsuca_odb12 database could also be identified in genomes of *M. meretrix* (n = 4401 (99.5%)) and *M. lamarckii* (n = 4402 (99.6%)), respectively. The BUSCO scores of *M. meretrix* and *M. lamarckii* genomes are comparable to the highest BUSCO score from the *M. petechialis* genome (n = 4403 (99.6%) and higher than other available *Meretrix* genomes (n ranging from 4263–4395 (96.4%–99.4%)). For genome annotations, BUSCO scores of 93.0% and 95.2% were attained from *M. meretrix* and *M. lamarckii* with the metazoa_odb12 database, and 94.3% and 96.3% with the mollsuca_odb12 database, respectively (Fig. 4B). The genome annotation BUSCO scores of *M. meretrix* and *M. lamarckii* from the mollsuca_odb12 database are comparable to that of *M. petechialis* (96.6%) and are higher than that of *Meretrix* sp. JML1 (73.8%), *Meretrix* sp. MT1 (84.8%) and *Meretrix* sp. MF1 (90.8%). Therefore, the high BUCO scores indicate high completeness of both genome assemblies and annotations of both *M. meretrix* and *M. lamarckii* among the available *Meretrix* genomes.

### Macrosynteny analysis

**Fig. 5:**
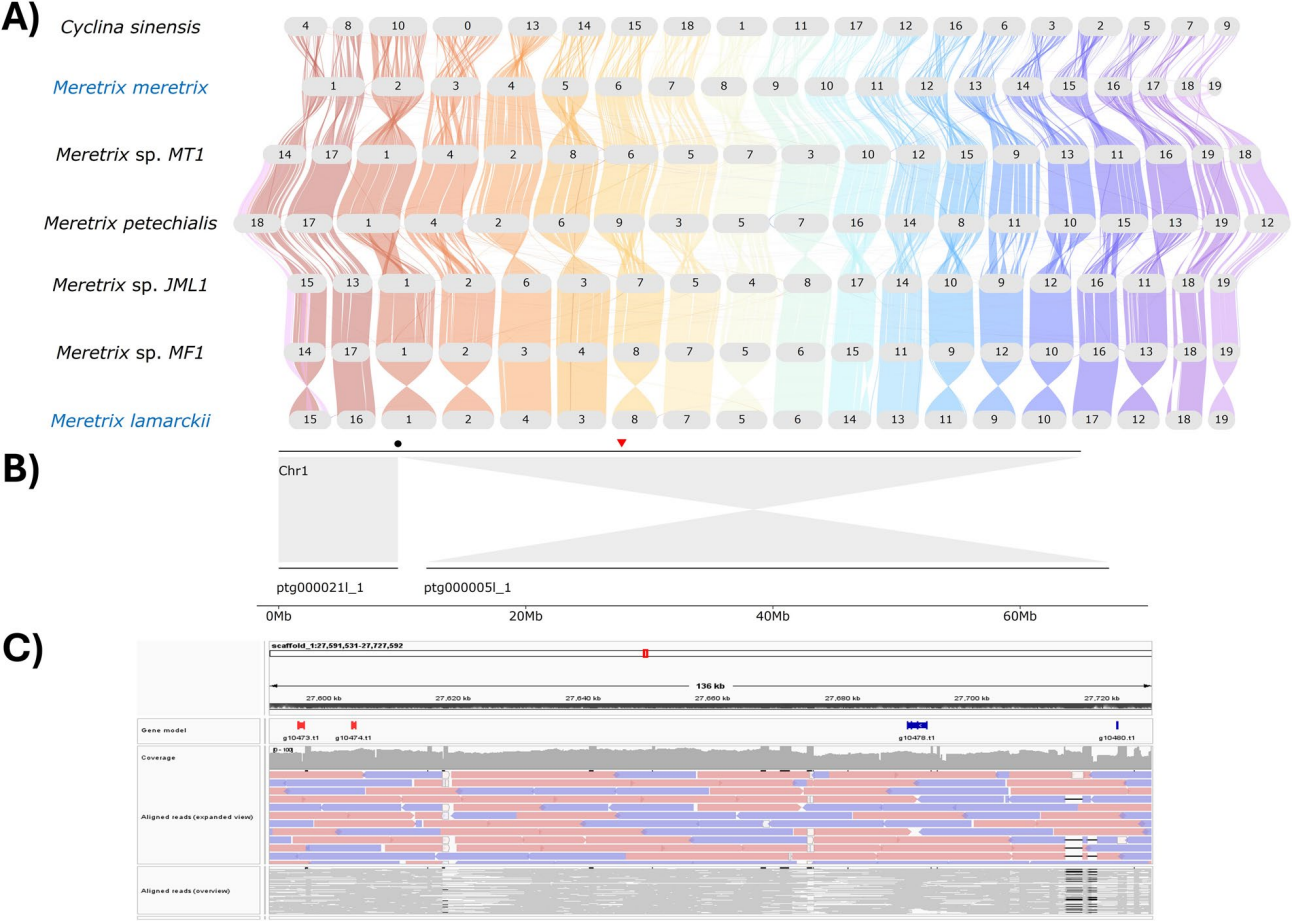
(**A**) Macrosynteny of *M. meretrix* and *M. lamarckii* and other published genomes; (**B**) Visualization of contigs assembled by Hifiasm aligned to Chr 1 in the final assembly of *M. meretrix*. Black circle denotes the assembly gap and red triangle indicates the genomic region of fusion/fission of Chr 1 in *M. meretrix*; (**C**) Visualization of PacBio HiFi reads aligned to the genomic region of fusion/fission of Chr 1 in *M. meretrix*. Genes have syntenic relationships to *M. lamarckii* Chr 15 and Chr 16 are coloured in red and blue, respectively. Under the expanded view of aligned reads, positive and negative strands of reads are coloured in pink and purple, respectively.

To validate the accuracy of the genome assemblies, the syntenic relationships of *M. meretrix* and *M. lamarckii* genomes were compared to other *Meretrix* genomes (*M. petechialis* (GCA_046203225.1)^10^, *Meretrix* sp. JML1 (GCA_049244375.1), *Meretrix* sp. MF1 (GCA_049244355.1) and *Meretrix* sp. MT1 (GCA_049244365.1)^7^ and *Cyclina sinensis* (GCA_012932295.1)^27^. The longest transcripts of gene sets of each genome were extracted for macrosynteny analysis using the GENESPACE pipeline^38^ with default parameters. Briefly, gene collinearity was detected by a minimum block size of 5 collinear genes using MCScanX^39^ and gene orthology inferred by OrthoFinder^26^ was used to define collinearity anchors. Macrosynteny analysis revealed a highly conserved chromosome synteny between the *Meretrix* species (Fig. 5A). While the *M. lamarckii* genome shared a one-to-one homologous relationship in all 19 chromosomes with other *Meretrix* species, chromosome fusion/fission events were also identified in *M. meretrix*, where chr1 corresponds to chr15 and chr16 of *M. lamarckii* and chr19 to a partial region of chr15 of *M. lamarckii*. For further validation, contigs assembled by Hifiasm were searched against the final assembly of *M. meretrix* using BLASTn. Chr1 was covered by two contigs with 100% identity match, namely “ptg000021l_1” and “ptg000005l_1”, where the latter encompasses the genomic region of chromosome fusion/fission (Fig. 5B). In addition, raw PacBio HiFi reads were mapped to the *M. meretrix* genome using minimap2^40^ and visualised in IGV (v2.19.5)^41^, and the region of chromosome fusion/fission were well supported by aligned PacBio HiFi reads with even and adequate coverage (>30×) (Fig. 5C), ruling out the possibility of a misassembled region.

## Data Records

The genome assemblies of *M. meretrix* and *M. lamarcki* are available at NCBI/ENA GenBank accessions JBTXPG000000000^42,43^ and JBTORC000000000^44,45^, DDBJ accessions PRJDB40269^46^ and PRJDB40270^47^, NGDC accessions GWHHOJO00000000^48^ and GWHHOOY00000000^49^, and CUHK Research Data Repository^50^, respectively. The raw sequencing data of *M. meretrix* and *M. lamarcki* are deposited in the NCBI/ENA database under the SRA accession numbers SRP617717^51,52^ and SRP618173^53,54^, DDBJ database under the DRA accession numbers DRR911404^55^-DRR911405^56^ and DRR911157^57^- DRR911158^58^, NGDC database under the GSA accession numbers CRA039134^59^ and CRA039136^60^, and CUHK Research Data Repository^50^, respectively. The genome annotation files of *M. meretrix* and *M. lamarckii* can be found at CUHK Research Data Repository^50^.

## Supplementary information

Supplementary Table

## Data Availability

The genome assemblies of *M. meretrix* and *M. lamarcki* are available at NCBI/ENA GenBank under accessions JBTXPG000000000 and JBTORC000000000, DDBJ accessions PRJDB40269 and PRJDB40270, NGDC accessions GWHHOJO00000000 and GWHHOOY00000000, and CUHK Research Data Repository (10.48668/DOVLYS), respectively. The raw sequencing data of *M. meretrix* and *M. lamarcki* are deposited in the NCBI/ENA database under the SRA accession numbers SRP617717 and SRP618173, DDBJ database under the DRA accession numbers DRR911404-DRR911405 and DRR911157-DRR911158, NGDC database under the GSA accession numbers CRA039134 and CRA039136, and CUHK Research Data Repository (10.48668/DOVLYS), respectively. The genome annotation files of *M. meretrix* and *M. lamarckii* can be found at CUHK Research Data Repository (10.48668/DOVLYS).

## Code availability

No specific script was used in this work.
